# Quality of clinical guidelines in pediatric headache

**DOI:** 10.1186/1824-7288-40-S1-A86

**Published:** 2014-08-11

**Authors:** Pasquale Parisi

**Affiliations:** 1 NESMOS Department, Chair of Paediatrics, Pediatric Headache Centre, Paediatric Sleep Centre & Child Neurology, Faculty of Medicine & Psychology, “Sapienza University”, Sant’Andrea Hospital, Via di Grottarossa, 1035-1039, Rome, Italy

## Background

Headache is a very common complaint in children, and can have a profound impact on school performance [1], being the major cause of absence from school [2], and interfering with other daily activities [3]. The studies based on parental reports may be an unreliable source of information on the frequency of headache in young children; in fact, it has been suggested that almost 36% of the parents of children with headache are unaware of the headache [4]. In any case, the increased incidence over the last 30 years probably reflects the significant changes in children’s lifestyles.

Given the elevated prevalence and the associated high degree of disability, it is not surprising that headache represents an important public health issue with considerable costs for the National Health Care System (NHCS), although, as children are not directly involved in the productivity process, it is not so easy to quantify the enormous, both, direct and indirect NHCS costs in this population [5].

## Methods

To assess the appropriateness and uniformity of application of the available pediatric clinical guidelines (CGs) for the diagnosis and treatment of headache in children, It has been conducted a systematic literature search using the following terms: headache, cephalalgia, guidelines and children (MESH or text words). Six CGs containing informations on the diagnosis and management of headache with specific recommendations for children were selected [6-11]. Eleven neuropediatric centers evaluated, by means of the AGREE II instrument, the quality and the appropriateness of available CGs.

## Results

NICE CGs resulted “strongly recommended”, while the French and Danish CGs were mainly “not recommended”. The comparison between the overall quality score of the French and NICE CGs was statistically significant (6.54 ± 0.69 vs 4.18 ± 1.08; p =0.001). A correlation analysis showed a significant association only for the “editorial independence” domain (r = 0.842 p = 0.035). The intra-class coefficients showed that the higher agreement between 11 reviewers was present for the Lewis CGs (r = 0.857) while the lower one for the NICE CGs (r = 0.656).

## Conclusions

CGs are definitely scarce and non "homogeneous". A major efforts to update the existing CGs according to principles of the evidence based medicine are needed.
